# MicroRNAs in Soft Tissue Sarcomas: Overview of the Accumulating Evidence and Importance as Novel Biomarkers

**DOI:** 10.1155/2014/592868

**Published:** 2014-08-04

**Authors:** Tomohiro Fujiwara, Toshiyuki Kunisada, Ken Takeda, Koji Uotani, Aki Yoshida, Takahiro Ochiya, Toshifumi Ozaki

**Affiliations:** ^1^Department of Orthopaedic Surgery, Okayama University Graduate School of Medicine, Dentistry, and Pharmaceutical Sciences, Okayama 7008558, Japan; ^2^Center for Innovative Clinical Medicine, Okayama University Hospital, Okayama 7008558, Japan; ^3^Department of Medical Materials for Musculoskeletal Reconstruction, Okayama University Graduate School of Medicine, Dentistry and Pharmaceutical Sciences, Okayama 7008558, Japan; ^4^Department of Intelligent Orthopaedic System, Okayama University Graduate School of Medicine, Dentistry and Pharmaceutical Sciences, Okayama 7008558, Japan; ^5^Division of Molecular and Cellular Medicine, National Cancer Center Research Institute, Tokyo 1040045, Japan

## Abstract

Sarcomas are distinctly heterogeneous tumors and a variety of subtypes have been described. Although several diagnostic explorations in the past three decades, such as identification of chromosomal translocation, have greatly improved the diagnosis of soft tissue sarcomas, the unsolved issues, including the limited useful biomarkers, remain. Emerging reports on miRNAs in soft tissue sarcomas have provided clues to solving these problems. Evidence of circulating miRNAs in patients with soft tissue sarcomas and healthy individuals has been accumulated and is accelerating their potential to develop into clinical applications. Moreover, miRNAs that function as novel prognostic factors have been identified, thereby facilitating their use in miRNA-targeted therapy. In this review, we provide an overview of the current knowledge on miRNA deregulation in soft tissue sarcomas, and discuss their potential as novel biomarkers and therapeutics.

## 1. Introduction

Sarcomas are malignant tumors of mesenchymal origin. Mesenchymal tissue is defined as a complex of nonepithelial structures of the body, which exclusively comprise the reproductive, glia, hematopoietic, and lymphoid tissues. The word “sarcoma” is derived from the Greek word sarkoma, meaning “fleshy outgrowth,” and can present as either a bone or soft tissue sarcomas [1]. Since the origin of soft tissue sarcomas has not been clarified, the classification system commonly used is based on histopathology. The world health organization (WHO) system is generally accepted as the basis for soft tissue tumor classification. According to the study based on the Surveillance, Epidemiology, and End Results (SEER), which included 26,758 cases from 1978 to 2001, leiomyosarcoma (LMS) was the most common form of sarcoma, accounting for 23% of all cases. Additional major histological types included in this study were malignant fibrous histiocytoma (MFH; 17%), liposarcoma (11%), dermatofibrosarcoma (10%), and rhabdomyosarcoma (RMS; 4%) [2]. Another report showed that MFH and LS are the most common types of soft tissue sarcomas in adults, accounting for 35%–45% of all sarcomas [3]. Notably, it is accepted that MHF does not show true histiocytic differentiation and its morphological pattern is shared by a variety of poorly differentiated malignancies. Accordingly, the diagnostic term MFH has been removed from WHO classification, and such lesions, without using the outdated terminology, are now included in the new category of undifferentiated/unclassified sarcomas.

Treatment options for most patients with sarcomas include surgical resection and adjuvant chemo- and radiotherapy. Despite the development of combined modality treatments in recent years, a significant proportion of patients with sarcomas respond poorly to chemotherapy, leading to local recurrence or distant metastasis. Lung metastasis is the main cause of death among patients with soft tissue sarcomas [4, 5]. Thus, early detection of recurrent or metastatic disease or early decision making according to tumor response to chemotherapy could improve patient prognosis. However, there are no useful biomarkers for these purposes. Indeed, only imaging methods are mostly used to detect or monitor tumor development. Thus, the discovery of novel biomarkers to detect tumors, predict their drug sensitivity, and monitor them is one of the most important challenges that must be overcome.

There is a growing amount of evidence in favor of utilizing miRNA profiling in the diagnosis of soft tissue sarcomas. Despite their small size (~22 nucleotides), these endogenous noncoding RNAs have an enormous effect on gene expression and regulate a variety of physiological and pathological processes [6–8]. Over the past several years, it has become evident that dysregulation of many types of miRNAs has been associated with the initiation and progression of human cancers [9]. A number of many studies have indicated that miRNAs can act as either oncogenes or tumor suppressors. The recent discovery of miRNAs as novel biomarkers in human serum or plasma has represented a new approach for the diagnostic screening for malignant diseases [8]. In addition, some successful* in vivo* studies support the concept that they may be used as innovative therapeutics to address unmet needs, although they are not presently used as cancer therapeutics [7].

In this review, we overview the accumulating evidence of miRNAs in soft tissue sarcomas, highlighting their function in each histological type of soft tissue sarcoma and their clinical relevance. Further, we update the clinical trials on the basis of miRNA profiling using patient blood samples as well as addressing the potential of miRNAs as novel biomarkers and therapeutics for soft tissue sarcomas.

## 2. Aberrant miRNA Expression in Soft Tissue Sarcomas (Table 1)

### 2.1. Liposarcoma

Liposarcoma is one of the most common soft tissue sarcomas in adults and can be subdivided into the following four major types: atypical lipomatous tumor/well-differentiated liposarcoma (WDLS), myxoid liposarcoma (MLS), pleomorphic liposarcoma (PLS), and dedifferentiated liposarcoma (DDLS). DDLS is defined as a WDLS that shows an abrupt transition to a nonlipogenic sarcoma. In addition to distinctive morphologies, each of the subgroups has a different prognosis and treatment strategy. MLS is relatively chemosensitive in comparison to the other types [10]. Although the prognosis of WDLS is good, that of DDLS is much worse, with a survival rate of approximately 28%–30% at the 5-year follow-up [11].

Most reports on miRNA profiling of liposarcoma have been specific to DDLS. Based on deep sequencing of small RNA libraries and hybridization-based microarrays, Ugras et al. identified more than 40 miRNAs that were dysregulated in DDLS and not in normal adipose tissue and WDLS. The upregulated miRNAs included miR-21 and -26, while the downregulated miRNAs included miR-143 and -145 [12]. Furthermore, reexpression of miR-143 in DDLS cell lines inhibited cell proliferation and induced apoptosis through downregulation of BCL2, topoisomerase 2A, protein regulator of cytokinesis 1 (PRC1), and polo-like kinase 1 (PLK1) [12]. A similar approach was adopted by Zhang et al., who performed miRNA profiling to compare WDLS/DDLS and normal adipose tissue. They determined that miR-155 was upregulated in DDLS, and silencing of miR-155 in DDLS cells inhibited cell growth and colony formation, induced G1-S cell-cycle arrest* in vitro*, and blocked tumor growth* in vivo* [13]. Further, they determined that miR-155 directly targeted casein kinase 1*α*, which enhanced *β*-catenin signaling [13]. Renner et al. identified miR-218-1∗ and HS_303_a as being upregulated miRNAs and miR-144 and -1238 as being downregulated miRNAs relative to that in normal adipose tissues [14]. Using unbiased genome-wide methylation sequencing, Taylor et al. identified that miR-193b was downregulated in DDLS relative to normal adipose tissue and WDLS, whose putative miR-193b promoters were differentially methylated [15]. A DDLS study by Hisaoka et al. focused on calreticulin (*CALR*), an inhibitor of adipocyte differentiation, and identified decreased expression of miR-1257, which targets* CALR* [16].

MLS has a unique genomic abnormality characterized by t(12; 16)(q13; p11) translocation, which creates the TLS-CHOP chimeric oncoprotein. Borjigin et al. investigated the molecular functions of TLS-CHOP and revealed that miR-486 was downregulated in both TLS-CHOP-expressing fibroblasts and MLS [17]. Since plasminogen activator inhibitor-1 (*PAI-1*) was identified as a target of miR-486, TLS-CHOP-miR-486-PAI-1 might be critical for MLS tumorigenesis and development [17]. In the miRNA profiling of MLS relative to normal adipose tissue, Renner et al. determined that miR-9, -891a, and -888 were upregulated and miR-486-3p and -1290 were downregulated. Interestingly, this was consistent with the report by Borjigin et al., who also reported on dysregulated miRNAs in PLS relative to normal adipose tissue and demonstrated that miR-1249, -296-5p, and -455-5p were upregulated and miR-200b∗, -200, and -139-3p were downregulated [14].

Recently published papers have demonstrated a clinical correlation with miRNA dysregulation and liposarcoma. In a single SNP array of 75 liposarcoma samples, Lee et al. identified frequent amplification of miR-26a-2c [18]. This miRNA was upregulated in not only WDLS/DDLS but also MLS. Importantly, high miR-26a-2 expression significantly correlated with poor patient survival in both types of liposarcoma, regardless of histological subtypes. An additional study revealed that the regulator of chromosome condensation and BTB domain-containing protein 1 (*RCBTB1*) was one of the targets of miR-26a-2, which regulates cellular apoptosis [18].

### 2.2. Rhabdomyosarcoma

RMS is not only the most common soft tissue sarcoma in children under 15 years of age (representing 5%–8% of all pediatric malignancies) but also one of the most common soft tissue sarcomas in adolescents and young adults [19]. Histopathologically, RMS is classified into the following four subtypes: embryonal RMS (ERMS), alveolar RMS (ARMS), pleomorphic RMS (PRMS), and spindle cell/sclerosing RMS. Most patients with RMS are treated with chemotherapy, and depending on the size and location of the primary tumor, most will also undergo either radiotherapy or surgery. Adult patients who showed complete response to chemotherapy had a 5-year survival rate of 57% compared to only 7% for poor responders [20].

Since RMS has been predicted to originate from mesenchymal progenitor cells located in muscle tissue, most studies have focused on miRNAs that are involved in skeletal muscle development (“muscle-specific miRNAs”) [21–23]. Global miRNA expression analysis was performed by Subramanian et al., which revealed that muscle-specific miRNAs (miR-1 and -133) were relatively downregulated in PRMS relative to normal skeletal muscle, and miR-335 was upregulated in ARMS relative to normal skeletal muscle [24]. miR-335 resides in intron 2 of* MEST*, which has been indicated to play a role in muscle differentiation. Furthermore, it shows high mRNA expression in ARMS. Notably,* MEST* is a downstream target of* PAX3*, the gene involved in the* PAX3-FKHR* fusion that is typical for ARMS. Rao et al. determined that miR-1 and -133a were drastically reduced in ERMS and ARMS cell lines [25]. Although these miRNAs affected cytostasis and differentiation in ERMS cells, this was not true for ARMS cells. Taulli et al. and Yan et al. examined the role of the muscle-specific miR-1 and -206 in RMS [26, 27]. They showed that their reexpression in RMS cells targeted* c-Met* mRNA to promote myogenic differentiation, decreased cell growth and migration, and inhibited tumor growth in xenografted mice. Furthermore, Li et al. reported on additional important targets. They showed that miR-1, -206, and -29 could regulate* PAX3* and* CCND2* expression [28]. Recently, Taulli et al. further pursued miR-206 targets. They focused on the BAF53a subunit of the SWI/SNF chromatin remodeling complex, which is an important molecule during myogenic differentiation. Indeed, the BAF53a transcript was present at significantly higher levels in primary RMS tumors compared with normal muscle. Silencing of BAF53a in RMS cells inhibited cell proliferation and anchorage-independent growth* in vitro*, inhibited ERMS and ARMS tumor growth, and induced myogenic differentiation* in vivo*, therefore, leading to the conclusion that failure to downregulate the BAF53a subunit may contribute to RMS pathogenesis [29].

Importantly, Missiaglia et al. demonstrated the clinical relevance of these muscle-specific miRNAs by using RT-PCR to investigate miR-1, -206, -133a, and -133b expression in 163 primary RMS samples [30]. The Kaplan-Meier curves showed a correlation between overall survival and miR-206 expression, whereas no correlation was observed with miR-1 or -133a/b. In particular, low miR-206 expression correlated with poor overall survival and was an independent predictor of shorter survival times in metastatic ERMS and ARMS cases without* PAX3/7-FOXO1* fusion genes [30]. Among the muscle-specific miRNAs, Ciarapica et al. found that miR-26a was also downregulated in RMS cells [31]. They further revealed that it may have a role in RMS pathogenesis via regulation of the expression of* Ezh2*, which regulates embryonic development through inhibition of homeobox gene expression [31]. miR-203 was also found to be downregulated in RMS by Diao et al. This occurred due to promoter hypermethylation and could be reexpressed by DNA-demethylating agents [32]. Reexpression of miR-203 suppressed tumor growth by directly targeting* p63* and* LIFR*, which lead to the inhibition of both the Notch and JAK1/STAT1/STAT3 pathways and promotion of myogenic differentiation [32].

Nonmuscle-specific miRNAs also have been reported as key molecules that function in RMS. Subramanian et al. showed that miR-29 was downregulated in RMS and acted as a tumor suppressor [24, 28, 33]. In the reports from Wang et al., NF-*κ*B and YY1 downregulation caused derepression of miR-29 during myogenesis, whereas, in RMS, miR-29 was epigenetically silenced by an activated NF-*κ*B-YY1 pathway. Reexpression of miR-29 in RMS inhibited tumor growth* in vivo* [33]. It has also been proposed that miR-29 can silence HDAC4 [34] or affect the Rybp epigenetic modifier [35], further promoting myogenic differentiation [21]. To date, HDAC inhibitors are promising agents for targeted therapy for metastatic RMS [36]. Sarver et al. reported that* EGR1* is regulated by miR-183 in multiple tumor types in addition to RMS, including synovial sarcoma and colon cancer [37]. Silencing of miR-183 in RMS cells revealed deregulation of a miRNA network composed of miR-183-EGR1-PTEN [37]. Armeanu-Ebinger et al. analyzed miRNA expression in ARMS and malignant rhabdoid tumor (MRT) in tissue samples and cell lines to identify their specific miRNA expression patterns. As a result, miR-9∗ was shown to be overexpressed in ARMS, whereas miR-200c was expressed at lower levels in ARMS than MRT [38]. Another important study on ARMS was reported by Reichek et al. They investigated the 13q31 amplicon that contains the miR-17-92 cluster gene and observed its significant overexpression in tumors with the 13q31 amplicon [39]. This was present in 23% of ARMS cases, especially in* PAX7-FKHR*-positive cases compared to* PAX3-FKHR*-positive and fusion-negative cases. Notably, high expression of the miR-17-91 cluster significantly correlated with poor prognosis in the 13q31-amplified group of patients, most of whom represented* PAX7-FKHR*-positive cases [39].

miRNA that is associated with drug resistant RMS has been reported. Chen et al. demonstrated that miR-485-3p was expressed at lower levels in drug-resistant lymphoblastic leukemia cells than in parental cells [40]. Facilitated by its promoter, miR-485-3p targets NF-YB, which may be a mediator of topoisomerase 2*α* [40]. They replicated these results in drug-sensitive and -resistant RMS cells and found that the miR-485-3p-Top2*α*-NF-YB pathway represented a general phenomenon associated with drug sensitivity.

### 2.3. Leiomyosarcoma

LMS is a malignant tumor showing smooth muscle differentiation. Soft tissue LMS usually occurs in middle-aged or older individuals, although it may develop in young adults and even in children [11]. It originates in retroperitoneal lesions (40%–45%), extremities (30%–35%), skin (15%–20%), and larger blood vessels (5%). Surgical resection is the most reliable treatment. Although the effectiveness of chemo- and radiotherapy is uncertain, a clear survival benefit of chemo- or radiotherapy is evident if surgical margins are not clear of tumor cells. For patients with LMS in the extremities, the reported local recurrence rate is 10%–25%, whereas the 5-year survival rate is 64% [41].

Accumulated studies on miRNA profiling of LMS have focused on those originating from the extremities and uterus. All studies have demonstrated upregulation of miRNAs in LMS relative to its benign counterparts such as leiomyoma or other soft tissue sarcomas. Subramanian et al. demonstrated that miR-1, -133a, and -133b, which play major roles in myogenesis and myoblast proliferation, are significantly overexpressed in LMS relative to normal smooth muscle [24]. Interestingly, miR-206, a miRNA that is highly expressed in normal skeletal muscle, was underexpressed in both LMS and normal smooth muscle [24]. Danielson et al. investigated miRNA profiling of uterine LMS and reported that the miR-17-92 cluster was overexpressed compared with myometrium [42]. Shi et al. focused on the overexpression of HMGA2 in uterine LMS and found that it is caused by let-7 repression [43]. Similarly, Nuovo et al. performed* in situ* hybridization and found that miR-221 was upregulated in uterine LMS but was not detected in leiomyomas or benign metastasizing leiomyomas [44]. Two recent reports have demonstrated miRNA dysregulation compared to the other sarcomas. Guled et al. profiled 10 high-grade LMS and 10 high-grade UPS samples with miRNA microarray and identified that miR-320a was upregulated in LMS relative to UPS [45]. In the examination of differentially expressed miRNAs in LMS compared to the other sarcoma subtypes, Renner et al. reported that miR-133a, -1, and -449a were upregulated, while miR-483-5p, -656, and -323-3p were downregulated [14]. These results were partly consistent with those of Subramanian et al. [24].

### 2.4. Synovial Sarcoma

Synovial sarcoma accounts for up to 10% of soft tissue sarcomas and includes two major histological subtypes, biphasic and monophasic [46]. They can occur anywhere in the body and feature local invasiveness and a propensity to metastasize [47]. Synovial sarcoma has a specific chromosomal translocation t(X; 18)(p11; q11) that leads to formation of an* SS18-SSX* fusion gene. Although treatment is based on surgery, adjuvant radio- or chemotherapy may be beneficial, particularly in high-risk patients. The 5-year overall survival is 55% for axial synovial sarcoma and 84% for extremity synovial sarcoma [47].

In the first report on miRNA profiling performed by Subramanian et al. in 2008, they utilized microarray, cloning, and northern blot analysis to demonstrate that miR-143 was downregulated in synovial sarcoma relative to GIST and LMS [24]. Since* SSX1* is predicted to be a target for miR-143 in* in silico* databases such as miRBase or TargetScan, it is speculated that its decreased expression in synovial sarcoma enables the production of the SS18-SSX1 oncoprotein. Sarver et al. focused on the molecular feature of synovial sarcoma that the SS18-SSX fusion protein represses* EGR1* expression through a direct association with the* EGR1* promoter. They investigated the correlation between* EGR1* and miR-183, which is significantly overexpressed in synovial sarcoma [37]. These studies found that miR-183 could target* EGR1* mRNA, which contributed to cell migration and invasion in synovial sarcoma cells. Through the functional analysis of many tumor cell lines, miR-183 was found to have an oncogenic role through the miR-183-EGR1-PTEN pathway in synovial sarcoma, RMS, and colon cancer [37]. Interestingly, Renner et al. also indicated that miR-183 is upregulated in synovial sarcoma relative to other sarcomas. Additional upregulated miRNAs demonstrating a >10-fold change were miR-200b∗ and -375, while the downregulated miRNAs showing >5.5-fold change included miR-34b∗, -142-5p, and -34c-3p [14]. Hisaoka et al. examined the global miRNA expression in synovial sarcoma and compared the results to Ewing sarcoma and normal skeletal muscle. Unsupervised hierarchical clustering revealed 21 significantly upregulated miRNAs, including let-7e, miR-99b, and -125-3p [48]. Functional analysis based on the silencing of let-7e and miR-99b resulted in the suppression of cell proliferation and the expression of* HMGA2* and* SMARCA5*, the putative targets of these miRNAs [48].

### 2.5. Malignant Peripheral Nerve Sheath Tumor

Malignant peripheral nerve sheath tumor (MPNST) typically originates from cells constituting the nerve sheath, such as Schwann and perineural cells. Approximately 50% of MPNSTs occur sporadically, with the remaining originating in patients with neurofibromatosis type 1 (NF1) [11]. Patients with NF1 have high risk of developing MPNSTs, and most are aggressive tumors with a poor prognosis.

Many reports have investigated the global miRNA profiling of MPNSTs in comparison with benign counterparts such as neurofibromas. Subramanian et al. determined the gene expression signature for benign and malignant peripheral nerve sheath tumors, which indicated that* p53* inactivation occurs in majority of MPNSTs [49]. They also performed miRNA profiling of these tumor sets and found a relative downregulation of miR-34a expression in most MPNSTs, concluding that* p53* inactivation and the subsequent loss of miR-34a expression may significantly contribute to MPNST development [49]. Itani et al. utilized a similar approach and identified the overexpression of miR-21 in MPNSTs compared to neurofibromas.* In silico* research predicted programmed cell death protein 4 (*PDCD4*) as a putative target of miR-21 [50]. Functional analysis using an MPNST cell line indicated that silencing of miR-21 could induce apoptosis of MPNST cells [50]. Presneau et al. also compared miRNA profiling between MPNSTs and NFs and identified 14 downregulated and 2 upregulated miRNAs. The former included miR-29c, -30c, -139-5p, 195, -151-5p, 342-5p, 146a, -150, and -223, and the latter included miR-210 and -339-5p [51]. Among them, miR-29c mimics reduced cell invasion of MPNST cells, regulating the expression of its target,* MMP2* [51]. Gong et al. identified the downregulated expression of miR-204 in MPNSTs in the same approach and reported* Ras* and* HMGA2* as the target molecules in MPNSTs [52]. Chai et al. utilized a different approach and found that miR-10b was upregulated in primary Schwann cells isolated from NF1 neurofibromas, and in cell lines and tumor tissues from MPNSTs [53]. Importantly, they showed that NF1 mRNA was the target for miR-10b. Zhang et al. focused on the expression of polycomb group protein enhancer of zeste homologue 2 (*Ezh2*), an important regulator for various human malignancies, and identified that it was significantly upregulated in MPNSTs [54]. Ezh2 inhibited miR-30d expression by binding to its promoter and an* in silico* database identified* KPNB1* as a miR-30d target. They concluded that EZH2-miR-30d-KPNB1 signaling was critical for MPNST survival and tumorigenicity [54].

### 2.6. Angiosarcoma

Angiosarcoma is a malignant tumor that recapitulates the morphological and functional characteristics of normal endothelium [11]. It accounts for less than 1% of all sarcomas and originates most commonly in the deep muscles of the lower extremities [3]. They are aggressive malignancies with a high rate of tumor-related death and more than half of all patients die within the first year [11].

In the web-accessible Sarcoma miRNA Expression Database (S-MED) generated by Sarver et al. [55], miRNAs that are significantly unregulated (>80-fold change) in angiosarcoma compared to other sarcomas included miR-520c-3p, -519a, and -520h (http://www.oncomir.umn.edu/). However, they have not been analyzed for their function in any cell lines. On the other hand, Italiano et al. investigated miRNA profiling based on* MYC* abnormalities in angiosarcoma. MYC amplification was identified in 3 out of 6 primary angiosarcomas and in 8 out of 12 secondary angiosarcomas by array-comparative genomic hybridization (aCGH) and FISH analysis. By comparing the miRNA profile of* MYC*-amplified and* MYC*-unamplified angiosarcomas using deep sequencing of small RNA libraries, they identified that the miR-17-92 cluster is preferentially overexpressed in* MYC*-amplified angiosarcoma. Since* MYC*-amplified angiosarcoma is associated with lower expression of thrombospondin-1 (*THBS1*),* MYC* amplification may be important in the angiogenic phenotype of angiosarcoma through upregulation of the miR-17-92 cluster, which downregulates* THBS1* expression [56].

### 2.7. Fibrosarcoma

Soft tissue fibrosarcoma is classified into infantile fibrosarcoma and adult fibrosarcoma. The infantile fibrosarcoma is histologically similar to classic adult fibrosarcoma but has a distinctive* ETV6-NTRK3* gene fusion and a favorable outcome. In contrast, >80% of adult fibrosarcoma cases were reported to be high-grade in the recent series of strictly defined cases [57].

To date, miRNA profiling has been limited to the fibrosarcoma cell line, HT1080. The first report came from Liu and Wilson, who investigated the correlation between matrix metalloproteinases (MMPs) and miR-520c and -373, which had been reported to play important roles in cancer cell metastasis as oncogenes [58]. Their data demonstrated that miR-520c and -373 suppressed the translation of* mTOR* and* SIRT1* by directly targeting the 3′-untranslated region (UTR). Since* mTOR* and* SIRT1* are negative regulators of* MMP9* via inactivation of the Ras/Raf/MEK/Erk signaling pathway and NF-*κ*B activity, these miRNAs were found to increase MMP9 expression by directly targeting* mTOR* and* SIRT1* and stimulating cell growth and migration [58]. Another investigation using HT1080 cells was reported by Weng et al., who focused on the regulatory mechanism of angiogenin (ANG) expression. In their* in silico* analysis, they found that* ANG* mRNA was targeted by miR-409-3p via its 3′UTR and overexpression of miR-409-3p in HT1080 cells silenced* ANG* expression [59]. Furthermore, their* in vitro* and* in vivo* analyses demonstrated that miR-409-3p inhibited tumor growth, vascularization, and metastasis via silencing* ANG* expression [59].

### 2.8. Undifferentiated Pleomorphic Sarcoma

In 2002, WHO declassified MFH as a formal diagnostic entity and renamed it as an undifferentiated pleomorphic sarcoma (UPS) not otherwise specified (NOS) [60]. In 2013, UPS/MFH was categorized in the undifferentiated/unclassified sarcomas [61]. Undifferentiated/unclassified sarcomas account for up to 20% of all sarcomas and have no clinical or morphological characteristics that would otherwise place them under specific types of sarcomas. Genetic subgroups are emerging within this entity.

Guled et al. conducted miRNA profiling on a series of LMS and UPS samples to identify specific signatures useful for differential diagnosis. They profiled 10 LMS and 10 UPS samples, using two cultured human mesenchymal stem cell samples as controls. As a result, 38 human miRNAs were determined to be significantly differentially expressed in UPS compared to control samples [45]. In UPS samples, miR-126, -223, -451, and -1274b were significantly upregulated (>2-fold change) and miR-100, -886-3p, -1260, -1274a, and -1274b were significantly downregulated (>3-fold change) compared to control samples [45]. When comparing the profiles of LMS and UPS, miR-199-5p was highly expressed in UPS, while miR-320a was highly expressed in LMS [45]. They also revealed that several genes, including IMP3, ROR2, MDM2, CDK4, and UPA, were targets of differentially expressed miRNAs and validated their expression in both sarcomas by immunohistochemistry.

### 2.9. Epithelioid Sarcoma

Epithelioid sarcoma represents between 0.6% and 1.0% of sarcomas and is most prevalent in adolescents and young adults between 10 and 35 years of age [62, 63]. This tumor is the most common soft tissue sarcoma in the hand and wrist, followed by ARMS and synovial sarcoma [3]. Two clinicopathological subtypes are recognized: (1) the conventional or classic (“distal”) form, characterized by its proclivity for acral sites and pseudogranulomatous growth pattern, and (2) the proximal-type (“large-cell”) variant that originates mainly in proximal/truncal regions and consists of nests and sheets of large epithelioid cells. The reported 5-year overall survival rates are 60%–80% [64–66] and the prognosis for patients with the proximal type is significantly worse than that for patients with the classic form [66–68].

Proximal-type epithelioid sarcoma has similarities with MRT, including the lack of nuclear immunoreactivity of* SMARCB1* (also known as* INI1*,* BAF47*, and* hSNF5*). Papp et al. hypothesized that miRNAs regulate* SMARCB1* expression and analyzed eight candidate miRNAs selected from* in silico* analysis. RT-PCR using tumor samples identified the overexpression of miR-206, -381, -671-5p, and -765 in epithelioid sarcomas [69]. Examination of the effect of miRNA transfections revealed that three of the overexpressed miRNAs (miR-206, miR-381, and miR- 671-5p) could silence* SMARCB1* mRNA expression in cell cultures. They concluded that the epigenetic mechanism of gene silencing by miRNAs caused the loss of* SMARCB1* expression in epithelioid sarcoma [69].

### 2.10. Kaposi's Sarcoma

Kaposi's sarcoma (KS) is the most common malignancy in untreated HIV-infected individuals. KS-associated herpesvirus (KSHV; also known as human herpesvirus 8) is the infectious cause of this neoplasm [70]. KSHV is a large DNA virus that encodes over 80 different proteins and is the causative agent of several diseases including not only KS but also the hyperproliferative B cell disorders, primary effusion lymphoma (PEL) and multicentric Castleman's disease [71]. Notably, recent discovery that KSHV encodes 12 miRNAs raises the possibility that these non-protein-coding gene products may contribute to viral-induced tumorigenesis [71–75].

Two groups have provided interesting evidence that KSHV-encoded miR-K-11 and miR-155 share a common set of mRNA targets (BACH-1, FOS, and LDOC-1) and binding sites; this finding implies a possible link between viral- and nonviral-mediated tumorigenesis [71, 76–78]. These are particularly interesting findings because miR-155 overexpression is associated with certain B cell lymphomas, raising the possibility that miR-K-11 expression may be one factor linking KSHV to B cell lymphoproliferative disease [78]. Other tumor-specific miRNAs have been reported by O'Hara et al. and Wu et al. O'Hara et al. profiled KS biopsies, PELs, normal tonsil tissue, and KSHV-infected and uninfected endothelial cells (ECs) because KS is a malignancy of ECs and is believed to be at the border between infection-induced hyperplasia and clonal neoplasia. As a result, multiple tumor suppressor miRNAs (miR-155, miR-220/221, and the let-7 family) are downregulated in KSHV-associated cancers, including PEL and KS [79]. Furthermore, they identified miR-143/145 as novel KS tumor-regulated miRNAs. Wu et al. also investigated a series of differentially expressed miRNAs and protein-coding genes associated with Kaposi's sarcomagenesis or KSHV infection. They found that the miR-221/222 cluster was downregulated, while miR-31 was upregulated in KS. Analysis of the putative miRNA targets revealed that ETS1 and ETS2 were downstream targets of miR-221/222, while FAT4 was one of the direct targets of miR-31 [80]. These molecules were involved in manipulating cell migration and motility. O'Hara et al. further analyzed pre-miRNA profiling of KS biopsies with well-established culture and mouse tumor models. As a result, increased miR-15 expression and decreased miR-221 demarked the malignant transition of endothelial cells, whereas increased miR-140 determined the degree of the transformation [81]. Interestingly, miR-24-2 pre-miRNA levels were strikingly elevated only in KS biopsies, thus, serving as a KS-specific biomarker [81].

### 2.11. Others

Greither et al. demonstrated a correlation of expression of a single miRNA with the age of tumor onset and the prognosis in a gender-specific manner in patients with soft tissue sarcomas. They focused on the expression levels of miR-210, a known hypoxia-regulated miRNA, since it is correlated with poor prognosis. In qRT-PCR analysis using the 78 tumor samples of soft tissue sarcomas, an intermediate expression of miR-210 was significantly correlated with poor prognosis of female patients with soft tissue sarcomas. They also found that miR-210 expression was significantly correlated with a 9.6-year later age of tumor onset in male patients with soft tissue sarcomas [82].

## 3. Comparison of Deregulated miRNAs in Bone Sarcomas and Soft Tissue Sarcomas

Extensive miRNA studies have been conducted on bone sarcomas such as osteosarcoma (OS), Ewing sarcoma, and chordoma [83–87]. Several deregulated miRNAs are commonly identified in soft tissue sarcomas and bone sarcomas, while several miRNAs are unique to their own histopathological classification of soft tissue sarcomas. Commonly upregulated miRNAs include miR-21 and the 17–92 cluster, whereas commonly downregulated miRNAs include miR-143, -1/206, -34a, and -100. miR-21 is upregulated in both DDLS and MPNST (Table 1) and also in OS [88]. miR-17-92 cluster is upregulated in ARMS, uterine LMS, angiosarcoma (Table 1), and in OS [89]. Indeed, these miRNAs are well-known oncomiRs that have also been identified in other cancers of the lung, stomach, esophagus, prostate, colon, ovaries, blood, pancreas, liver, and breasts [90–92]. Therefore, miR-21 and the miR-17-92 cluster have been considered to be representative oncomiRs for a wide variety of malignant neoplasms. On the other hand, miR-143 is commonly downregulated in DDLS, SS (Table 1), and OS [93], while miR-34a is downregulated in MPNST, OS, and Ewing sarcoma [86, 94]. These miRNAs are also widely reported as tumor-suppressor miRNAs in a variety of cancers such as breast, lung, colon, kidney, bladder, and skin cancer. Indeed, miR-34a is a direct transcriptional target of p53 [95], a central tumor suppressor, and p53 enhances the posttranscriptional maturation of several miRNAs with growth-suppressive function, including miR-16-1, miR-143, and miR-145, in response to DNA damage [96]. Therefore, miR-34a and -143 are classified as representative tumor suppressor miRNAs for a variety of malignancies including bone and soft tissue sarcomas. It is interesting that muscle-specific miR-1/206 is downregulated in RMS and chordoma [97], but the molecular mechanisms of miR-1/206 downregulation in chordoma have not been elucidated.

miRNAs that are unique in their histology include miR-26a in DDLS and miR-203 in RMS (Table 1). To date, their deregulation have not been identified in other soft tissue sarcomas or bone sarcomas. Indeed, miR-26a has been reported as a key miRNA in adipocyte differentiation. Indeed, miR-26a has been reported as a key miRNA in adipocyte differentiation [18, 98], whereas miR-203 suppresses p63 and LIFR, which in turn leads to the downregulation of the Notch pathway and the LIFR-dependent JAK1/STAT1/STAT3 pathway [99]. These pathways are indispensable for the maintenance and proliferation of muscle satellite cells during normal muscle development and muscle regeneration, and also inhibits myogenic differentiation by repressing MEF2 and MyoD [100, 101]. Thus, these results indicate that the deregulation of miRNAs that correlate with the differentiation of normal cells and tissues may play an important role in tumorigenesis of mesenchymal origin.

## 4. Challenge for the Clinical Application of miRNA as a Novel Biomarker

Emerging reports have demonstrated that circulating miRNAs are useful for tumor detection. To date, studies on breast, colon, prostate, and ovarian cancers have shown the possibilities of circulating miRNAs as diagnostic and prognostic markers for each cancer [102–105]. The first report of circulating miRNAs as potential diagnostic markers in sarcomas was presented in 2010 [106]. To date, the studies on soft tissue sarcoma have been reported in two histological types [107]: RMS and MPNST (Table 2).

### 4.1. Rhabdomyosarcoma

The first trial of circulating miRNAs as novel biomarkers in sarcomas was performed using serum samples derived from patients with RMS. Miyachi et al. focused on muscle-specific miRNAs (miR-1, -133a, -133b, and -206) that were shown to be more abundantly expressed in myogenic tumors [106]. Expression levels of these muscle-specific miRNAs in RMS cell lines were analyzed and, compared to those in neuroblastoma, Ewing sarcoma, and MRT cell lines, miR-206 was most abundantly expressed in RMS cells. Notably, these results were reflected in culture supernatants of RMS cell lines. They also confirmed that muscle-specific miRNAs were significantly upregulated in RMS tumor specimens. In their analysis of muscle-specific miRNA serum levels in patients with RMS and without RMS, serum levels of these miRNAs were significantly higher in the former. Among these miRNAs, normalized serum miR-206 showed the highest sensitivity and specificity among muscle-specific miRNAs [106]. Importantly, miR-206 expression levels decreased after RMS treatment compared to the pretreatment condition. This result was consistent with the evidence based on the previous studies using RMS tissues [26, 27, 30], indicating that miRNA deregulation in patient tissue specimens could reflect those in patient serum.

### 4.2. Malignant Peripheral Nerve Sheath Tumor

A recent report from Weng et al. has shown the possibility of miRNAs representing novel, noninvasive biomarkers for the diagnosis of MPNST. They performed genome-wide serum miRNA expression analysis in order to distinguish MPNST patients with and without NF1. Solexa sequencing was applied to screen for differentially expressed miRNAs in pooled serum from 10 patients with NF1, 10 patients with sporadic MPNST, and 10 patients with NF1 and MPNST. On the basis of validation studies on more patient sets, miR-801 and -214 showed higher expression in patients with sporadic MPNST and patients with NF1 and MPNST than patients with NF1 [108]. In addition, miR-24 was significantly upregulated only in patients with NF1 and MPNST. Therefore, they concluded that the combination of the three miRNAs (miR-801, -214, and -24) could distinguish patients with sporadic MPNST from those with NF1 and MPNST [108].

## 5. Conclusions and Future Directions

Sarcomas are distinctly heterogeneous tumors of mesenchymal origin [4, 84, 109, 110]. More than 100 sarcoma subtypes have been described [11]; however, this variety can present a diagnostic challenge because their clinical and histopathological characteristics are not always distinct [111]. In these past three decades, genetic exploration has greatly improved the diagnosis for soft tissue sarcomas, including the identification of fusion genes in soft tissue sarcomas such as synovial sarcoma, MLS, or clear cell sarcoma. The identification of miRNAs specific to histological subtypes may be a novel breakthrough for sarcoma research. As shown in Tables 1 and 2, a variety of miRNAs have been detected by various approaches. These miRNAs include those related to chromosomal translocation of each subtype or those associated with the cell differentiation of the normal counterpart. An important step forward has been achieved on the basis of miRNA research for further understanding of sarcomagenesis and sarcoma development.

To date, there are few useful biomarkers to monitor tumor development, which is one of the important problems in soft tissue sarcomas. However, several researchers have shown the possibility of miRNAs as novel biomarkers for monitoring sarcomas or for their differential diagnosis using patient-derived serum or plasma. Since these trials of “liquid biopsy” have been limited to a few histological subtypes, further exploration to include a variety of subtypes is expected. In addition, there is no evidence for miRNAs serving as biomarkers that reflect drug resistance. These miRNAs would help clinicians to determine the optimal individual treatment options, thus leading to the improvement of the patients' prognosis. Another problem is that there are not a few cases that cannot be classified into the current histological classification. In such cases, miRNA profiling may help in obtaining a differential diagnosis or creating a novel category of histopathological classification.

Emerging reports indicate the possibility of “miRNA therapeutics” in bone sarcomas. For example, supplementary administration of miR-143 mimic or miR-133a inhibitor into osteosarcoma-bearing mice using conventional chemotherapy has been shown to inhibit osteosarcoma lung metastasis [84, 93]. We have now identified some* in vivo* trials for soft tissue sarcomas, most of which utilize viral transduction into cells prior to xenografting into mice, while few trials have utilized systemic administration of oligonucleotide. The high number of mRNAs targeted by a single miRNA may represent an advantage compared to specific gene silencing by siRNA. Notably, this method also means that each miRNA can modulate several molecular pathways with potentially unpredictable side effects. Identification of the miRNAs that are critical and specific to each sarcoma (among the reported miRNAs as shown in Table 1) would be an important step to the clinical application of “miRNA therapeutics.”

**Figure 1:**
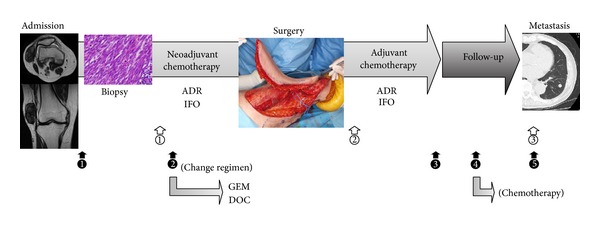
Examples of clinical applications of miRNAs as biomarkers and therapeutics for patients with soft tissue sarcoma. As therapeutics: ① combination with neoadjuvant chemotherapy, ② combination with adjuvant chemotherapy, and ③ combination with chemotherapy for metastasis. As biomarkers: *❶* diagnosis, *❷* determination of drug resistance, *❸* monitoring after treatment for primary lesions, *❹* detection for micrometastasis, and *❺* monitoring after treatment for metastasis. ADR: adriamycin; IFO: ifosfamide; GEM: gemcitabine; DOC: docetaxel.

While some issues remain unresolved regarding the monitoring of circulating miRNA as biomarkers or the efficacy of miRNA delivery, novel trials for noninvasive miRNA-based diagnosis and for highly efficacious “miRNA therapeutics” will be a worthwhile step for clinical applications in the near future (Figure 1).

## Figures and Tables

**Table 1:** Deregulated miRNAs in soft tissue sarcomas.

Histology	miRNAs	Expression level	Function	miRNA target	Reference
Liposarcoma	miR-21, -26a (DDLS)	Increased	N/D	N/D	[12]
	miR-143, -145 (DDLS)	Decreased	Cell proliferation, apoptosis	*BCL2, Topoisomerase 2A, PRC1, and PLK1 *	[12]
	miR-155 (DDLS)	Increased	Cell proliferation, colony formation, and tumor growth	*CK1* *α*	[13]
	miR-218-1∗ (DDLS)	Increased	N/D	N/D	[14]
	miR-144, -1238 (DDLS)	Decreased	N/D	N/D	[14]
	miR-193b (DDLS)	Decreased	N/D (methylated)	N/D	[15]
	miR-1257 (DDLS)	Decreased	N/D	*CALR *	[16]
	miR-486 (MLS)	Decreased	Cell proliferation	*PAI-1 *	[17]
	miR-486-3p, -1290 (MLS)	Decreased	N/D	N/D	[14]
	miR-9, -891a, and -888 (MLS)	Increased	N/D	N/D	[14]
	miR-1249, -296-5p, and -455-5p (PLS)	Increased	N/D	N/D	[14]
	miR-200b∗, -200, and -139-3p (PLS)	Decreased	N/D	N/D	[14]
	miR-26a-2 (DDLS, MLS)	Increased	Clonogenicity, adipocyte differentiation, and cell apoptosis	*RCBTB1 *	[18]

Rhabdomyosarcoma	miR-1, -133a/b	Decreased	Myogenic differentiation, cell proliferation	*SRF, Cyclin D2 *	[23, 25]
	miR-206	Decreased	Myogenic differentiation, cell growth, cell migration, tumor growth, and correlation with prognosis	*c-Met, PAX3, PAX7, CCDN2, HDAC4, and BAF53a *	[23, 25–30]
	miR-26a	Decreased	N/D	*Ezh2 *	[31]
	miR-203	Decreased	Myogenic differentiation, cell proliferation, cell migration, and tumor growth	*p63, LIFR *	[32]
	miR-335 (ARMS)	Increased	N/D	*CHFR, HAND1, SP1 *	[24]
	miR-29	Decreased	Cell cycle arrest, muscle differentiation, tumor growth	*YY1 *	[28, 32]
	miR-183	Increased	Cell migration, and cell invasion	*EGR1, PTEN *	[37]
	miR-9∗	Increased	Cell migration	*E-cadherin *	[38]
	miR-200c	Decreased	Cell migration	N/D	[38]
	miR-17-92 cluster (ARMS)	Increased	Correlation with prognosis in 13q31 amplified ARMS	N/D	[39]
	miR-485-3p	N/D	Drug resistance	*NF-YB *	[40]

Leiomyosarcoma	miR-1, -133a, and -133b	Increased	N/D	N/D	[24]
	miR-17-92 cluster (uterine LMS)	Increased	Smooth muscle differentiation	N/D	[42]
	let-7 (uterine LMS)	N/D	Cell proliferation	*HMGA2 *	[43]
	miR-221 (uterine LMS)	Increased	N/D	N/D	[44]
	miR-320a	Increased	N/D	N/D	[45]
	miR-133a, -1, and -449a	Increased	N/D	N/D	[14]
	miR-483-5p, -656, and -323-3p	Decreased	N/D	N/D	[14]

Synovial sarcoma	miR-143	Decreased	N/D	*SSX1 *	[24]
	miR-183	Increased	Cell migration, cell invasion	*EGR1 *	[37]
	let-7e, miR-99b, miR-125a-3p	Increased	Cell proliferation	*HMGA2, SMARCA5 *	[48]
	miR-200b∗, -183, and -375	Increased	N/D	N/D	[14]
	miR-34b∗, -142-5p, and -34c-3p	Decreased	N/D	N/D	[14]

MPNST	miR-34a	Decreased	Apoptosis	*MYCN, E2F2, and CDK4 *	[49]
	miR-10b	Increased	Cell proliferation, migration, and invasion	*NF1 *	[53]
	miR-21	Increased	Apoptosis	*PDCD4 *	[50]
	miR-204	Increased	Cell proliferation, migration, and invasion	*HMGA2 *	[52]
	miR-29c	Decreased	Cell invasion	*MMP2 *	[51]
	miR-210, -339-5p	Increased	N/D	N/D	[51]
	miR-30d	Decreased	Apoptosis	*KPNB1 *	[54]

Angiosarcoma	miR-520c-3p, -519a, and -520h	Increased	N/D	N/D	[55]
	miR-17-92 cluster (*myc*-amplified AS)	Increased	N/D	*THBS1 *	[56]

Fibrosarcoma	miR-520c, -373	N/D	Cell growth, cell migration	*mTOR, SIRT1 *	[58]
	miR-409-3p	N/D	Cell proliferation, tumor growth, vascularization, and metastasis	*ANG *	[59]

UPS	miR-126, -223, -451, and -1274b	Increased	N/D	N/D	[45]
	miR-100, -886-3p, -1260, -1274a, and -1274b	Decreased	N/D	*IMP3 *	[45]

Epithelioid sarcoma	miR-206, -381, and -671-5p	Increased	N/D	*SMARCB1 (INI1) *	[69]

Kaposi's sarcoma	miR-155, -K12-11	N/D	N/D	*BACH-1, FOS, and LDOC-1 *	[71, 76, 77]
	miR-155, -220/221, let-7	Decreased	Transition to tumorigenic endothelial cells	N/D	[79]
	miR-221/-222	Decreased	Cell migration	*ETS1, ETS2 *	[80]
	miR-31	Increased	Cell migration	*FAT4 *	[80]
	miR-15, 140	Increased	Transition to tumorigenic endothelial cells	N/D	[81]
	miR-24-2	Increased	N/D	N/D	[81]

Soft tissue sarcomas	miR-210	N/D	Correlates with age of tumor onset (male) and prognosis (female)	N/D	[82]

DDLS: dedifferentiated liposarcoma; MLS: myxoid liposarcoma; PLS: pleomorphic liposarcoma; LMS: leiomyosarcoma; ARMS: alveolar rhabdomyosarcoma; AS: angiosarcoma; MPNST: malignant peripheral nerve sheath tumor; UPS: undifferentiated pleomorphic sarcoma; N/D: no data.

**Table 2:** Studies on circulating miRNAs in the serum of patients with soft tissue sarcomas.

Histology	Promising circulating miRNAs	Study design	Samples	Sample size	Methods	Number of miRNAs examined	Normalization	References
Rhabdomyosarcoma	miR-206	RMS versus non-RMS versus healthy individual	Serum	8 RMS patients versus 23 non-RMS patients versus 17 healthy controls	qRT-PCR	4 miRNAs	miR-16	[106]

Malignant peripheral nerve sheath tumor	miR-24, 801, and 214	Sporadic MPNST versus NF1 MPNST versus NF1	Serum	(Screening) 10 sporadic MPNSTs versus 10 NF1 MPNSTs versus 10 NF1	Solexa sequencing, qRT-PCR	Genome-wide profiling	cel-miR-39	[108]
				(Validation) 83 sporadic MPNSTs versus 61 NF1 MPNSTs versus 90 NF1				

RMS: rhabdomyosarcoma; MPNST: malignant peripheral nerve sheath tumor; NF1: neurofibromatosis type 1.

## Abbreviations

WDLS: Well-differentiated liposarcoma
MLS: Myxoid liposarcoma
DDLS: Dedifferentiated liposarcoma
RMS: Rhabdomyosarcoma
ARMS: Alveolar rhabdomyosarcoma
ERMS: Embryonal rhabdomyosarcoma
LMS: Leiomyosarcoma
MPNST: Malignant peripheral nerve sheath tumor
MFH: Malignant fibrous histiocytoma
MRT: Malignant rhabdoid tumor
UPS: Undifferentiated pleomorphic sarcoma.
